# Renal Schwannoma: A Rare Case Report and Literature Review

**DOI:** 10.7759/cureus.32236

**Published:** 2022-12-05

**Authors:** Garrett A Britt, Henry Mroch, Allison M Young, Shane M Pearce

**Affiliations:** 1 Department of Urology, Washington State University Elson S. Floyd College of Medicine, Spokane, USA; 2 Department of Nephrology, Washington State University Elson S. Floyd College of Medicine, Spokane, USA; 3 Department of Pathology, Incyte Diagnostics, Spokane, USA; 4 Department of Urology, Spokane Urology, Spokane, USA

**Keywords:** retroperitoneal tumor, renal mass, schwannoma, renal, kidney

## Abstract

Schwannomas originating in the kidney are extremely rare with very few cases documented in the literature. It is difficult to distinguish them from other common renal masses based on clinical symptoms and imaging characteristics alone, as both are non-specific for this pathology. Thus, the final diagnosis of schwannoma is typically made only after surgical resection and histologic examination. We present the case of a 66-year-old female who was initially evaluated for flank pain and referred to us after a renal mass was found on CT imaging. A partial nephrectomy was performed, and subsequent pathological examination confirmed the diagnosis of renal schwannoma.

## Introduction

Schwannomas are benign neoplasms that originate from Schwann cells within the peripheral nerve sheath. Most occur sporadically as solitary tumors while others have a genetic predisposition and arise in association with neurofibromatosis type 2, schwannomatosis, or Carney’s complex [1]. Schwannomas are generally slow-growing and may exist asymptomatically for years before presenting with non-specific symptoms depending on anatomical location [2]. They are most commonly found in the head and neck, which accounts for 25-45% of all schwannoma cases [3,4]. Of those, approximately 57% arise from the vestibulocochlear nerve [5]. Retroperitoneal involvement is less common and accounts for only three percent of schwannomas [4]. However, schwannomas originating within the kidney are extremely rare. The first case of this neoplasm manifesting as a renal mass was documented in 1955 [6]. Since then, less than 40 cases have been described in the English literature, and this exact number has been variable and inconsistently reported. Here, we present the case of a patient diagnosed with renal schwannoma following resection and pathologic examination of a renal mass in the setting of non-specific urologic symptoms. We also present an updated review of this pathology’s presence in the English literature.

## Case presentation

A healthy 66-year-old female presented for evaluation of new-onset right-sided flank pain and bilateral lower extremity edema of one-month duration. The flank pain was sharp, non-radiating, and not associated with a colicky component. She denied additional urologic symptoms including dysuria, discolored urine, hematuria, foaming of her urine, as well as any constitutional symptoms. She endorsed a 15-pack-year smoking history, having quit 24 years prior, and she denied exposure to industrial solvents.

On initial presentation, her blood pressure was 153/77 mmHg with a pulse of 67 beats per minute, and her BMI was 29 kg/m^2^. Physical examination revealed a pleasant female appearing her stated age. Examination of her head and neck demonstrated normal hearing acuity without palpable cervical lymphadenopathy. Cardiovascular and respiratory examinations were unremarkable. Inspection of her lower extremities revealed bilateral 1+ edema to the mid-calf level without any bullous changes or skin lesions on her legs. Examination of her abdomen by inspection and palpation did not demonstrate distension, tenderness, or signs of a mass. Percussion to her bilateral flanks did not elicit any tenderness. 

**Figure 1:**
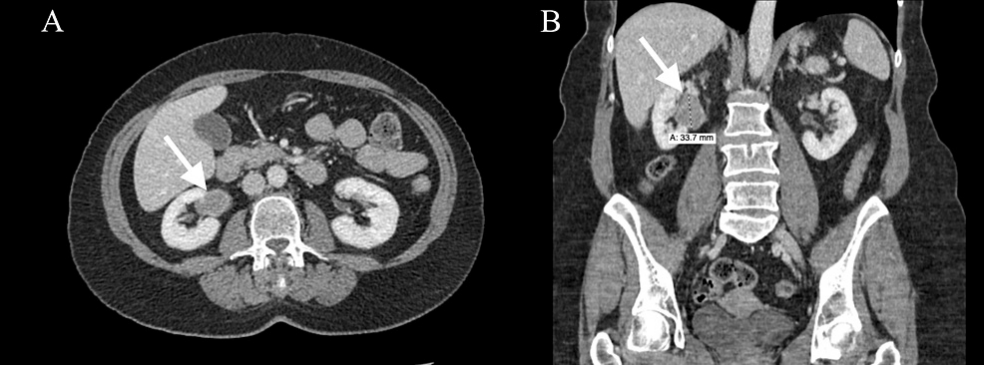
Representative CT of right renal mass (A) Axial and (B) coronal views showing a 3.4 cm tumor abutting the renal pelvis (solid white arrow).

Initial laboratory studies demonstrated a complete blood count within normal limits, serum creatinine of 0.68 mg/dL, potassium of 3.9 mEq/L, and bicarbonate of 23 mEq/L. Her urinalysis was negative for heme pigment and protein, and with a specific gravity of 1.020. Computed tomography (CT) of the abdomen and pelvis with radiocontrast revealed a 3.4 x 2.3 x 3.0 cm enhancing soft tissue mass in the right inferior pole of the renal pelvis directly abutting the renal vein and hilum without hydronephrosis (Figures 1A, 1B). No metastatic foci or involvement of the renal vein were observed. 

**Figure 2:**
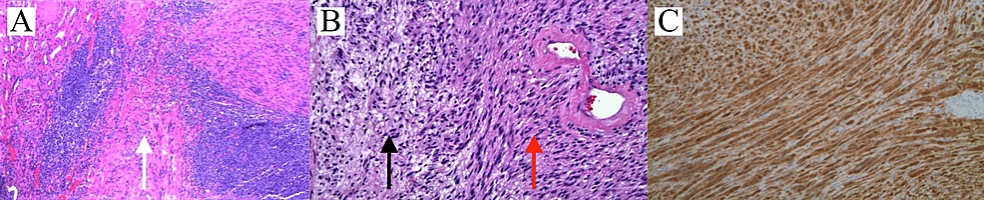
Histopathology of the resected mass (A) Low-power hematoxylin and eosin (H&E, x4) stained image shows palisading spindle cells (solid white arrow) of the schwannoma with renal parenchyma on the left. (B) Intermediate power (H&E, x10) shows a hypercellular area (Antoni A) on the right (solid red arrow) and a hypocellular area (Antoni B) on the left (solid black arrow). (C) Strong, diffusely positive S-100 immunostain (x10).

Laparoscopic robotic-assisted partial nephrectomy was performed which revealed a mass located at the hilum, inferior to the renal vein and abutting the inferior pole and ureter. The tumor was well encapsulated and successfully resected with negative margins. There were no intra-operative complications. Gross examination of the tumor was described as tan-brown in color and approximately 3.5 x 3.3 x 1.2 cm and without adherent adipose tissue. The capsular surface was intact and there was no gross evidence of invasion of adherent adipose tissue. Hematoxylin and eosin (H&E) histologic examination revealed a tumor consisting of well-demarcated spindle cells organized in both hypercellular and hypocellular patterns. Immunostaining for S-100 was positive (Figures 2A-2C).

Together, these findings supported the diagnosis of schwannoma. No post-operative complications were noted at subsequent follow-up appointments, and the patient’s initial presenting symptoms have improved. She will continue to be monitored with routine surveillance imaging for signs of tumor recurrence.

## Discussion

Schwannomas typically arise in the head, neck, and upper extremities. While retroperitoneal involvement is uncommon, renal schwannomas are extremely rare, with very few cases documented in the literature [4]. The first case was documented in 1955, and since then existing literature reviews have identified approximately 34 cases [6]. However, recent reports documenting renal schwannoma cases present varying, inconsistent statistics that may not accurately reflect the current literature. A PubMed search of the English literature, in addition to this case, instead revealed a total of 48 cases (Table 1). However, it is likely that this number is higher when cases of renal schwannoma published in non-English literature are considered. 

**Table 1:** Recorded cases of renal schwannoma in the English literature NA: Not available

Author, Year	Age (yrs)	Sex	Symptoms	Side	Location	Size (cm)	Treatment
Present case, 2022	66	F	Flank pain, lower extremity edema	R	Hilum	3.5	Nephrectomy
Dahmen et al., 2021 [2]	47	M	Flank pain	R	NA	12	Nephrectomy
Vidal Crespo et al., 2020 [6]	66	M	Incidental finding	R	Parenchyma	3.5	Nephrectomy
Wang et al., 2020 [7]	56	F	Flank pain	L	Hilum	11.5	Nephrectomy
Madueke et al., 2019 [8]	62	F	Incidental finding	L	Parenchyma	2.5	Nephrectomy
Iannaci et al., 2016 [9]	56	M	Back pain, hematuria	L	Parenchyma	4	Nephrectomy
Kelley et al., 2016 [10]	43	F	Abdominal pain	R	Hilum	4.3	Nephrectomy
Herden et al., 2015 [11]	69	F	Hematuria	R	Pelvis	6.5	Tumor excision
Yong et al., 2015 [12]	55	F	Abdominal pain, hematuria	R	Hilum	5.1	Nephrectomy
Kumano et al., 2015 [13]	73	M	NA	R	Hilum	3.5	Tumor excision
Hanashima et al., 2015 [4]	63	F	Incidental finding	L	Hilum	2.4	Tumor excision
Verze et al., 2014 [14]	59	M	Incidental finding	R	Parenchyma	15	Nephrectomy
Wang et al., 2013 [15]	66	M	Hematuria	L	Parenchyma	2.7	Nephrectomy
Mikkilineni et al., 2013 [16]	36	F	Flank pain, fever	R	Parenchyma	4.6	Tumor excision
Yang et al., 2012 [17]	40	F	Flank pain	L	Pelvis	6.8	Nephrectomy
Nayyar et al., 2011 [18]	44	F	Flank pain	R	Hilum	12	NA
Sfoungaristos et al., 2011 [19]	55	F	Incidental finding	L	Hilum	2.8	Nephrectomy
Raju et al., 2010 [20]	38	F	Incidental finding	L	Parenchyma	3.6	Nephrectomy
Qiguang et al., 2010 [21]	34	M	Hematuria	R	Hilum	2.6	Tumor excision
Gobbo et al., 2008 [22]	35	F	Flank and abdominal pain, nausea	L	Hilum	7	Nephrectomy
Gobbo et al., 2008 [22]	27	F	Incidental finding	R	Parenchyma	8.5	Nephrectomy
Gobbo et al., 2008 [22]	59	F	Incidental finding	L	Hilum	4.8	Nephrectomy
Hung et al., 2008 [23]	36	F	Palpable mass, flank pain	L	Parenchyma	7	Nephrectomy
Umphrey et al., 2007 [24]	63	F	Hot flashes, hypertension	R	Parenchyma	7	Nephrectomy
Tokunaga et al., 2005 [25]	39	F	Incidental finding	R	Hilum	8	Nephrectomy
Singh et al., 2005 [26]	35	M	Flank pain, gross hematuria	R	Pelvis	NA	Nephrectomy
Singh et al., 2005 [26]	40	M	Renal colicky pain, vomiting	L	Hilum	3	Nephrectomy
Cachay et al., 2003 [27]	74	F	Incidental finding	R	Capsule	9	Nephrectomy
Tsurusaki et al., 2001 [28]	69	F	Incidental finding	L	Capsule	NA	Tumor excision
Alvarado-Cabrero et al., 2000 [29]	45	M	Flank and abdominal pain	L	Parenchyma	16	Nephrectomy
Alvarado-Cabrero et al., 2000 [29]	40	F	Flank pain, abdominal mass	L	Parenchyma	12.5	Nephrectomy
Alvarado-Cabrero et al., 2000 [29]	84	M	Incidental finding	R	Parenchyma	4	Nephrectomy
Alvarado-Cabrero et al., 2000 [29]	18	F	Flank pain	R	Parenchyma	6.2	Nephrectomy
Pantuck et al., 1996 [30]	50	F	Palpable mass	R	Capsule	28	Nephrectomy
Singer et al., 1996 [31]	70	F	Incidental findings	L	Hilum	6	Nephrectomy
Bezzi et al., 1996 [32]	60	M	NA	R	Hilum	NA	NA
Ikeda et al., 1996 [33]	89	M	Abdominal pain	R	Pelvis	NA	Nephrectomy
Romics et al., 1992 [34]	52	M	Back and flank pain	R	Capsule	NA	Nephrectomy
Naslund et al., 1991 [35]	50	F	Abdominal pain, weight loss	L	Parenchyma	14	Nephrectomy
Inoue et al., 1991 [36]	51	F	Hematuria	L	Hilum	NA	Nephrectomy
Kitagawa et al., 1990 [37]	51	M	Abdominal pain, fever	L	Hilum	2.8	Nephrectomy
Ma et al., 1990 [38]	67	M	Epigastric pain	R	Parenchyma	8	Nephrectomy
Somers et al., 1988 [39]	55	F	Incidental findings	L	Parenchyma	5.1	Nephrectomy
Steers et al., 1985 [40]	50	F	Palpable mass	R	Hilum	9	Tumor excision
Bair et al., 1978 [41]	56	M	Microhematuria	R	Hilum	7	Nephrectomy
Fein et al., 1965 [42]	51	F	Pyelonephritis, palpable mass	R	Pelvis	6	Nephrectomy
Kuz’mina et al., 1962 [43]	33	F	Palpable mass, fever	R	Capsule	NA	Nephrectomy
Phillips et al., 1955 [44]	56	M	Flank pain	L	Pelvis	12	Nephrectomy

Renal schwannomas are slow-growing, benign tumors presenting with non-specific symptoms [2]. As a result, they are often diagnosed incidentally [20]. Abdominal and flank pain are the most common presenting symptoms while gross hematuria and fever are less common [2]. Existing literature suggests that renal schwannomas primarily originate in the renal hilum (44%) followed by the parenchyma (31%) and capsule [2]. Age at diagnosis is variable, with a range of 18-89 years and a median of 53 years (Table 1). The average tumor size is approximately 7.3 cm. Renal schwannomas have a female predominance, with 63% of cases being found in women. 

While CT and magnetic resonance imaging (MRI) are the primary imaging modalities used, renal schwannomas cannot easily be diagnosed or differentiated from other renal masses using imaging findings alone [6]. Renal schwannomas have been shown on MRI to be isointense on T1-weighted images and hyperintense on T2-weighted images [2,6] Likewise, with gadolinium contrast, there is a homogenous enhancement of the solid component of renal schwannomas on T1-weighted images. However, these MRI findings are non-specific, and renal schwannomas are often misdiagnosed as renal cell carcinoma until pathologic examination can be done to confirm the diagnosis either through resection or biopsy. With the exception of to select few cases, CT-guided needle biopsy is generally not used given limited accuracy [2,7]. It is difficult to make the diagnosis with confidence using a needle biopsy given the tumor’s rare presentation in the kidney.

Schwannomas are encapsulated and well circumscribed. On histologic examination, schwannomas consist of spindle cells with eosinophilic cytoplasm and elongated nuclei arranged in Antoni A and Antoni B patterns [6]. Antoni A refers to a dense, hypercellular arrangement of cells often with Verocay bodies, which are characterized by palisading nuclei surrounding cytoplasmic processes [2,6]. Antoni B refers to a looser, hypocellular arrangement of cells with a myxoid component [2,6]. Renal schwannomas are also positive for S-100 staining, which is indicative of tumors arising from neural crest cells [2,4]. The majority of renal schwannomas are benign, with only four reported cases of malignant transformation [4,7]. 

Given that renal schwannomas are usually benign and slow-growing, surveillance for well-selected patients is a common approach. Surgical resection with either a partial or radical nephrectomy is preferred if intervention is warranted based on the size and location of the tumor [2,7]. Partial nephrectomy has previously been described and allows for tumor excision with preservation of functional renal parenchyma optimizing kidney function [4]. Often, they are excised due to suspicion for renal cell carcinoma due to their rarity and the appearance of a solid enhancing mass on radiographic imaging [2]. For these reasons, surgical resection remains the primary method of treatment [6].

## Conclusions

Renal schwannomas are rare solid tumors of the kidney. It is suspected that the true number of renal schwannomas has been underestimated and inconsistently reported in the literature given the confounding findings often suggestive of renal cell carcinoma. This report presents a case of renal schwannoma and offers a current and comprehensive account of this condition in the English literature. Renal schwannomas pose a diagnostic challenge using clinical exam findings, laboratory studies, or imaging studies alone. As a result, renal cell carcinoma is often suspected initially, and the final diagnosis of schwannoma is dependent upon surgical resection and histologic examination. Thus, it is important to consider the diagnosis of schwannoma as a possibility when investigating any renal mass.
